# Crystal structure of (1*S*,3*R*,8*R*,10*S*)-2,2-di­chloro-10-hy­droxy-3,7,7,10-tetra­methyl­tri­cyclo­[6.4.0.0^1,3^]dodecan-9-one

**DOI:** 10.1107/S2056989016006174

**Published:** 2016-04-15

**Authors:** Ahmed Benzalim, Aziz Auhmani, My Youssef Ait Itto, Jean-Claude Daran, Auhmani Abdelwahed

**Affiliations:** a Laboratoire de Physico-Chimie Moléculaire et Synthèse Organique, Département de Chimie, Faculté des Sciences, Semlalia BP 2390, Marrakech 40001, Morocco; b Laboratoire de Chimie de Coordination, CNRS UPR8241, 205 route de Narbonne, 31077 Toulouse Cedex 04, France

**Keywords:** crystal structure, α-hy­droxy­ketone, asymmetric synthesis, natural products, absolute configuration, hydrogen bonding

## Abstract

The asymmetric unit of the title compound, C_16_H_24_Cl_2_O_2_, contains two independent mol­ecules (*A* and *B*) which are built from three fused rings, *viz.* a seven-membered heptane ring, a six-membered cyclo­hexyl ring bearing a ketone and an alcohol group, and a cyclo­propane ring bearing two Cl atoms. In the crystal, the two mol­ecules are linked *via* two O—H⋯O hydrogen bonds, forming an *A*–*B* dimer with an *R*_2_^2^(10) ring motif. The *A* mol­ecules of these dimers are linked *via* a C—H⋯O hydrogen bond, forming chains propagating along the *a*-axis direction. Both mol­ecules have the same absolute configuration, *i.e.* 1*S*,3*R*,8*R*,10*S*, which is based on the synthetic pathway and further confirmed by resonant scattering [Flack parameter = 0.03 (5)].

## Abstract 2

The asymmetric unit of title compound contains two independent mol­ecules which are built from three fused rings: a heptane ring, a cyclo­hexyl ring bearing a ketone and an alcohol group, and a three-membered cyclo­propane ring bearing two Cl atoms. In the crystal, the mol­ecules are linked by O—H⋯O and C—H⋯O hydrogen bonds, forming chains propagating along [100].

## Chemical context   

α-Hy­droxy carbonyl groups are present in many compounds (such as α-ketols) with important biological activity (Murahashi *et al.*, 1993 ▸). The hy­droxy­ketone side chain is not just found in a large variety of anti-inflammatory corticosteroid drugs (Van Rheenen & Shephard, 1979 ▸), but is also a structural component of adriamycin, a potent anti­tumor agent (Tamura *et al.*, 1985 ▸). As a result of their expanded occurrence and their biological activity, the development of methods for the direct asymmetric synthesis of α-hy­droxy ketones has grown significantly (Salvador *et al.*, 2006 ▸). In a tentative attempt to prepare new α-hy­droxy ketones with a natural product skeleton, we synthesized the title compound by oxidative ring-opening of (1*S*,3*R*,8*S*,9*R*,10*S*)-2,2-di­chloro-3,7,7,10-tetra­methyl-9,10-ep­oxy­tri­cyclo­[6.4.0.0^1,3^]dodecane (Sbai *et al.*, 2002 ▸), using aqueous CrO_3_ (Trost & Fray, 1988 ▸).
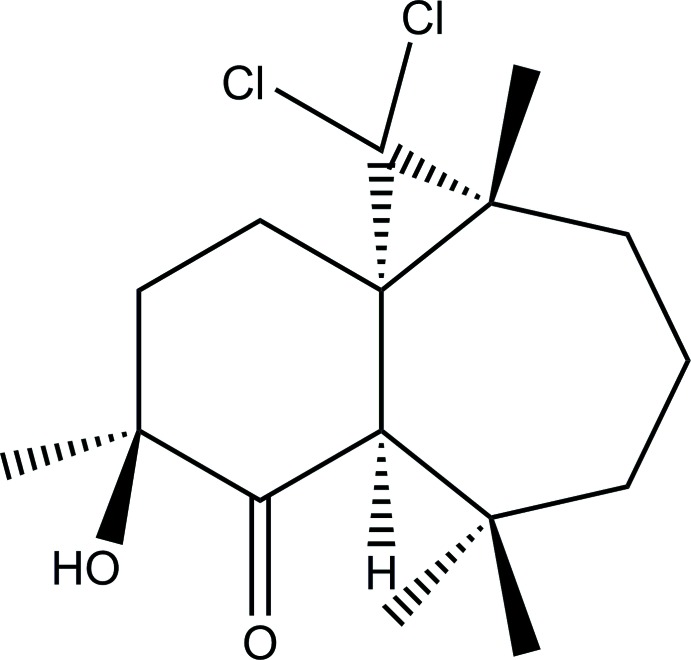


## Structural commentary   

**Figure 1:**
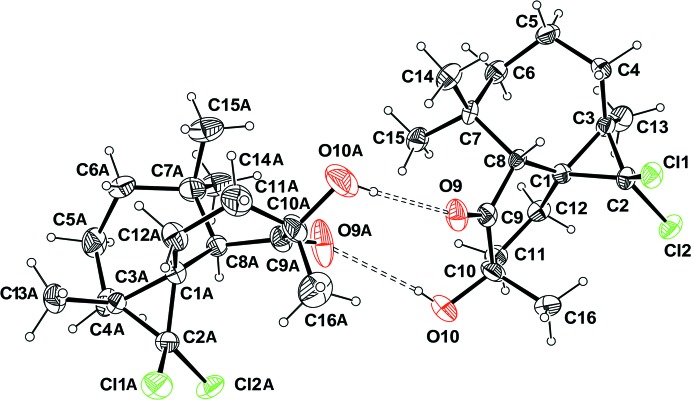
A view of the mol­ecular structure of the two independent mol­ecules of the title compound, showing the atom labelling. Displacement ellipsoid are drawn at the 50% probability level.

**Figure 2:**
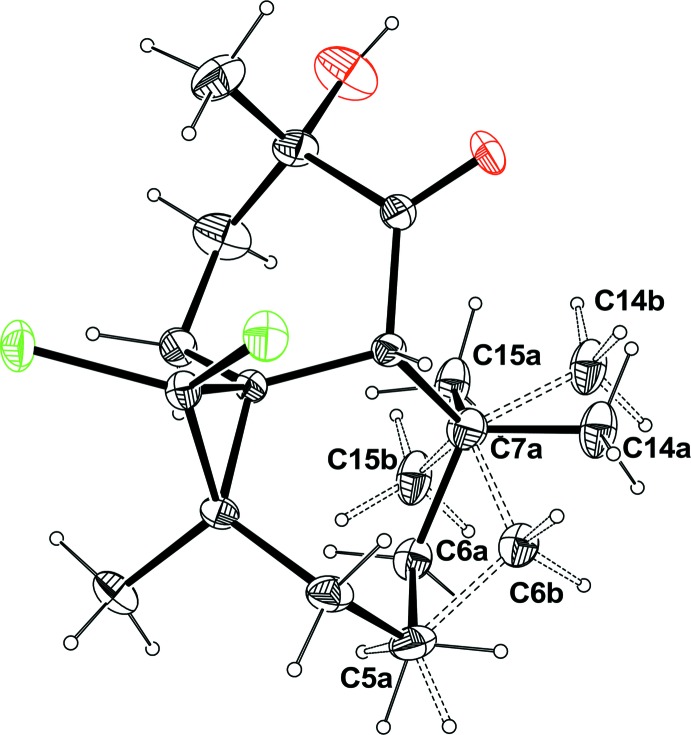
A view showing the disorder (dashed double lines) in mol­ecule *B*.

There are two mol­ecules (*A* and *B*) in the asymmetric unit of the title compound, Fig. 1 ▸, both having the same the absolute configuration: (1*S*,3*R*,8*R*,10*S*) and (1*AS*,3*AR*,8*AR*,10*AS*). The compound is built up from three fused rings: a seven-membered heptane ring, a six-membered cyclo­hexyl ring bearing a ketone and alcohol groups, and a three-membered propane ring bearing two Cl atoms (Fig. 1 ▸). In mol­ecule *B* (Fig. 2 ▸), there is positional disorder affecting the location of atom C6 which is split over two positions, C6*a* and C6*b*. In both mol­ecules, the six-membered rings display a conformation inter­mediate between boat and twist-boat with puckering parameters θ = 89.73 and φ2 = 198.07° for mol­ecule *A* and θ = 91.78 and φ2 = 210.97° for mol­ecule *B*. The seven-membered cyclo­heptane ring in mol­ecule *A* displays a conformation inter­mediate between boat and twist-boat with puckering parameters *q*2 = 1.151 (5) and *q*3 = 0.030 (5) Å. Owing to the disorder observed in mol­ecule *B* within the seven-membered ring, the conformation of this ring is inter­mediate between boat and twist-boat [*q*2 = 1.194 (5), *q*3 = 0.00 (4) Å] or chair and twist-chair [*q*2 = 0.363 (5), *q*3 = 0.784 (5) Å], depending on the position of atom C6*a* or C6*b*.

## Supra­molecular features   

**Figure 3:**
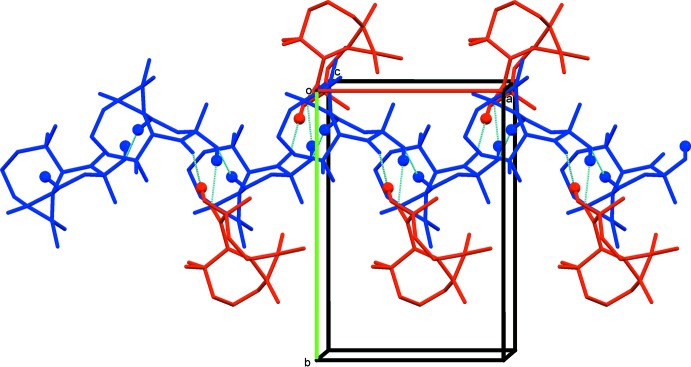
Partial crystal packing of the title compound (mol­ecule *A* blue, mol­ecule *B* red), viewed along the *c* axis, showing the formation of the hydrogen-bonded chain parallel to the *a*-axis direction. The hydrogen bonds are shown as dashed lines (see Table 1 ▸; H atom as balls) and H atoms not involved in these inter­actions have been omitted for clarity.

The two independent mol­ecules are connected through O—H⋯O hydrogen bonds, involving the hydroxyl and the ketone O atoms, forming an A-B dimer with an 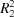
(10) ring motif (Fig. 3 ▸ and Table 1 ▸). The A mol­ecules of these dimers are linked via a C—H⋯O hydrogen bond forming chains propagating along the *a* axis direction (Fig. 3 ▸ and Table 1 ▸).

## Database survey   

A search of the Cambridge Structural Database (CSD, Version 5.38, update February 2016; Groom *et al.*, 2016 ▸) using a fused cyclo­hexyl, cyclo­heptane and cyclo­propane bearing two Cl atoms, the same main skeleton as in the title compound, revealed the presence of eight structures with similar cyclo­heptane rings. One of these concerns the starting reagent (XOSFUG; Sbai *et al.*, 2002 ▸) for the synthesis of the title compound – see Section 5. *Synthesis and crystallization*. In another compound, the cyclo­heptane ring is fused with a cyclo­hexane ring bearing a ketone group, *viz*. (1*S*,3*R*,8*S*,10*R*)-2,2-di­chloro-3,7,7,10-tetra­methyl­tri­cyclo­(6.4.0.01,3)dodec-9-one (XOSGAN; Sbai *et al.*, 2002 ▸). A search for a cyclo­hexa­n­one ring revealed the occurrence of one structure having a similar hy­droxy cyclo­hexa­none ring, *viz.* 6-(2-(3,4-dihy­droxy-4-methyl­cyclo­hex­yl)prop-2-en-1-yl)-2-hy­droxy-2-methyl-5-(prop-1-en-2-yl)cyclo­hexa­none monohydrate (BUXNAK; Blair *et al.*, 2010 ▸).

## Synthesis and crystallization   

To a solution of 0.4 g (1.319 mmol) of (1*S*,3*R*,8*S*,9*R*,10*S*)-2,2-di­chloro-3,7,7,10-tetra­methyl-9,10-ep­oxy­tri­cyclo­[6.4.0.0^1,3^]dodecane (Sbai *et al.*, 2002 ▸) in acetone (8 ml), 3 ml of an aqueous solution of CrO_3_ (1 g, 10 mmol) was added at 273 K. The mixture was stirred at room temperature for 30 min and cooled to 273 K in an ice bath and 1.5 ml of an aqueous solution of CrO_3_ (0,5 g, 5 mmol) was added dropwise. The ice bath was removed and the mixture was stirred at room temperature for 1 h. The reaction mixture was extracted with di­chloro­methane (3 × 30 ml) and the organic layers were dried over anhydrous Na_2_SO_4_ and the solvent was removed under reduced pressure. The crude product was then purified on silica gel chromatography (230–400 mesh) using hexa­ne/ethyl acetate (95:5) as eluent to give the title compound (yield 53%). Colourless plate-like crystals were obtained from a petroleum ether solution, by slow evaporation of the solvent at room temperature.

## Refinement   

Crystal data, data collection and structure refinement details are summarized in Table 2 ▸. The OH and C-bound H atoms were included in calculated positions and refined as riding: O—H = 0.84, C—H = 0.98–1.00 Å with *U*_iso_(H) = 1.5*U*_eq_(O and C-meth­yl) and 1.2*U*_eq_(C) for other H atoms. The disordered cyclo­heptane ring in mol­ecule *B* was refined by splitting atoms C6*a*, C14*a* and C15*a* over two positions. The occupancy factors were initially refined and once the occupancy was correctly evaluated the values were held fixed with ratio 0.54:0.46. Atoms C5*a* and C7*a* were also split (C5*a*/C5*b* and C7*a*/C7*b*) and constrained to occupy the same site using EXYZ and EADP commands allowing then to locate the H atoms.

## Supplementary Material

Crystal structure: contains datablock(s) I, Global. DOI: 10.1107/S2056989016006174/su5293sup1.cif

Structure factors: contains datablock(s) I. DOI: 10.1107/S2056989016006174/su5293Isup2.hkl

Supporting information file. DOI: 10.1107/S2056989016006174/su5293Isup3.cml

CCDC reference: 1473671

Additional supporting information:  crystallographic information; 3D view; checkCIF report

## Figures and Tables

**Table 1:** Hydrogen-bond geometry (Å, °)

*D*—H⋯*A*	*D*—H	H⋯*A*	*D*⋯*A*	*D*—H⋯*A*
O10—H10⋯O9*A*	0.84	2.43	3.203 (7)	153
O10*A*—H10*A*⋯O9	0.84	2.11	2.945 (6)	173
C12—H12*B*⋯O10^i^	0.99	2.48	3.361 (7)	148

Symmetry code: (i) 
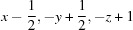
.

**Table 2:** Experimental details

Crystal data	
Chemical formula	C_16_H_24_Cl_2_O_2_
*M*_r_	319.25
Crystal system, space group	Orthorhombic, *P*2_1_2_1_2_1_
Temperature (K)	173
*a*, *b*, *c* (Å)	9.6745 (3), 13.9432 (6), 23.3654 (10)
*V* (Å^3^)	3151.8 (2)
*Z*	8
Radiation type	Mo *K*α
μ (mm^−1^)	0.41
Crystal size (mm)	0.45 × 0.35 × 0.10

Data collection	
Diffractometer	Agilent Xcalibur (Eos, Gemini ultra)
Absorption correction	Multi-scan (*CrysAlis PRO*; Agilent, 2014 ▸)
*T*_min_, *T*_max_	0.974, 1.000
No. of measured, independent and observed [*I* > 2σ(*I*)] reflections	16637, 5991, 5182
*R*_int_	0.062
(sin θ/λ)_max_ (Å^−1^)	0.617

Refinement	
*R*[*F* ^2^ > 2σ(*F* ^2^)], *wR*(*F* ^2^), *S*	0.052, 0.134, 1.07
No. of reflections	5991
No. of parameters	376
No. of restraints	12
H-atom treatment	H-atom parameters constrained
Δρ_max_, Δρ_min_ (e Å^−3^)	0.73, −0.46
Absolute structure	Flack *x* determined using 1835 quotients [(*I* ^+^)−(*I* ^−^)]/[(*I* ^+^)+(*I* ^−^)] (Parsons *et al.*, 2013 ▸)
Absolute structure parameter	0.03 (5)

Computer programs: *CrysAlis PRO* (Agilent, 2014 ▸), *SIR97* (Altomare *et al.*, 1999 ▸), *ORTEP-3 for Windows* (Farrugia, 2012 ▸), *Mercury* (Macrae *et al.*, 2008 ▸), *SHELXL2014* (Sheldrick, 2015 ▸) and *PLATON* (Spek, 2009 ▸).

## supplementary crystallographic information

### Crystal data

**Table:**

C_16_H_24_Cl_2_O_2_	*D*_x_ = 1.346 Mg m^−^^3^
*M**_r_* = 319.25	Mo *K*α radiation, λ = 0.71073 Å
Orthorhombic, *P*2_1_2_1_2_1_	Cell parameters from 6322 reflections
*a* = 9.6745 (3) Å	θ = 3.7–27.0°
*b* = 13.9432 (6) Å	µ = 0.41 mm^−^^1^
*c* = 23.3654 (10) Å	*T* = 173 K
*V* = 3151.8 (2) Å^3^	Plate, colourless
*Z* = 8	0.45 × 0.35 × 0.10 mm
*F*(000) = 1360	

### Data collection

**Table:**

Agilent Xcalibur (Eos, Gemini ultra) diffractometer	5991 independent reflections
Radiation source: Enhance (Mo) X-ray Source	5182 reflections with *I* > 2σ(*I*)
Graphite monochromator	*R*_int_ = 0.062
Detector resolution: 16.1978 pixels mm^-1^	θ_max_ = 26.0°, θ_min_ = 3.1°
ω scans	*h* = −11→11
Absorption correction: multi-scan (*CrysAlis PRO*; Agilent, 2014)	*k* = −15→17
*T*_min_ = 0.974, *T*_max_ = 1.000	*l* = −28→27
16637 measured reflections	

### Refinement

**Table:**

Refinement on *F*^2^	Hydrogen site location: inferred from neighbouring sites
Least-squares matrix: full	H-atom parameters constrained
*R*[*F*^2^ > 2σ(*F*^2^)] = 0.052	*w* = 1/[σ^2^(*F*_o_^2^) + (0.0594*P*)^2^ + 0.805*P*] where *P* = (*F*_o_^2^ + 2*F*_c_^2^)/3
*wR*(*F*^2^) = 0.134	(Δ/σ)_max_ < 0.001
*S* = 1.07	Δρ_max_ = 0.73 e Å^−^^3^
5991 reflections	Δρ_min_ = −0.46 e Å^−^^3^
376 parameters	Absolute structure: Flack *x* determined using 1835 quotients [(*I*^+^)-(*I*^-^)]/[(*I*^+^)+(*I*^-^)] (Parsons *et al.*, 2013)
12 restraints	Absolute structure parameter: 0.03 (5)

### Special details

**Table:**

Geometry. All esds (except the esd in the dihedral angle between two l.s. planes) are estimated using the full covariance matrix. The cell esds are taken into account individually in the estimation of esds in distances, angles and torsion angles; correlations between esds in cell parameters are only used when they are defined by crystal symmetry. An approximate (isotropic) treatment of cell esds is used for estimating esds involving l.s. planes.

### Fractional atomic coordinates and isotropic or equivalent isotropic displacement parameters (Å^2^)

**Table:**

	*x*	*y*	*z*	*U*_iso_*/*U*_eq_	Occ. (<1)
C1	0.0491 (5)	0.0997 (4)	0.40650 (19)	0.0207 (10)	
C2	0.0427 (5)	−0.0041 (4)	0.3866 (2)	0.0229 (11)	
C3	−0.0896 (5)	0.0502 (4)	0.3921 (2)	0.0240 (10)	
C4	−0.1630 (5)	0.0827 (4)	0.3381 (2)	0.0273 (12)	
H4A	−0.2365	0.0360	0.3286	0.033*	
H4B	−0.0962	0.0836	0.3061	0.033*	
C5	−0.2276 (5)	0.1830 (4)	0.3446 (2)	0.0305 (12)	
H5A	−0.2402	0.2114	0.3062	0.037*	
H5B	−0.3202	0.1764	0.3623	0.037*	
C6	−0.1411 (5)	0.2509 (4)	0.3809 (2)	0.0284 (12)	
H6A	−0.1397	0.2251	0.4204	0.034*	
H6B	−0.1903	0.3131	0.3824	0.034*	
C7	0.0096 (5)	0.2716 (3)	0.3636 (2)	0.0226 (11)	
C8	0.0986 (5)	0.1750 (3)	0.36409 (19)	0.0192 (10)	
H8	0.0893	0.1460	0.3251	0.023*	
C9	0.2505 (5)	0.1979 (4)	0.3721 (2)	0.0249 (11)	
C10	0.3245 (5)	0.1663 (4)	0.4267 (2)	0.0315 (12)	
C11	0.2258 (6)	0.1708 (4)	0.4777 (2)	0.0313 (13)	
H11A	0.2087	0.2390	0.4871	0.038*	
H11B	0.2719	0.1412	0.5111	0.038*	
C12	0.0859 (5)	0.1211 (4)	0.46868 (19)	0.0255 (11)	
H12A	0.0861	0.0600	0.4902	0.031*	
H12B	0.0126	0.1622	0.4852	0.031*	
C13	−0.1889 (5)	0.0269 (4)	0.4410 (2)	0.0353 (13)	
H13A	−0.2373	−0.0332	0.4327	0.053*	
H13B	−0.2564	0.0789	0.4451	0.053*	
H13C	−0.1367	0.0201	0.4767	0.053*	
C14	0.0144 (6)	0.3136 (4)	0.3030 (2)	0.0328 (13)	
H14A	−0.0431	0.3714	0.3014	0.049*	
H14B	−0.0206	0.2662	0.2757	0.049*	
H14C	0.1100	0.3302	0.2933	0.049*	
C15	0.0628 (6)	0.3474 (4)	0.4049 (2)	0.0310 (12)	
H15A	0.0163	0.4085	0.3973	0.046*	
H15B	0.1627	0.3553	0.3998	0.046*	
H15C	0.0438	0.3273	0.4443	0.046*	
C16	0.3835 (6)	0.0654 (4)	0.4177 (3)	0.0396 (14)	
H16A	0.4486	0.0662	0.3856	0.059*	
H16B	0.3080	0.0207	0.4093	0.059*	
H16C	0.4316	0.0447	0.4525	0.059*	
O9	0.3149 (4)	0.2414 (3)	0.33530 (17)	0.0364 (9)	
O10	0.4396 (4)	0.2273 (3)	0.4373 (2)	0.0477 (11)	
H10	0.4380	0.2736	0.4143	0.072*	
Cl1	0.10699 (13)	−0.03802 (9)	0.31897 (5)	0.0289 (3)	
Cl2	0.07611 (14)	−0.09878 (9)	0.43518 (6)	0.0331 (3)	
C1A	0.6252 (5)	0.6364 (4)	0.3446 (2)	0.0236 (11)	
C2A	0.7685 (5)	0.6191 (4)	0.3695 (2)	0.0249 (11)	
C3A	0.7087 (5)	0.7175 (4)	0.3728 (2)	0.0235 (11)	
C4A	0.6605 (6)	0.7556 (4)	0.4303 (2)	0.0332 (12)	
H4A1	0.6504	0.7010	0.4571	0.040*	
H4A2	0.7327	0.7986	0.4459	0.040*	
C5A	0.5252 (6)	0.8098 (4)	0.4280 (3)	0.0408 (14)	0.54
H5A1	0.5457	0.8791	0.4241	0.049*	0.54
H5A2	0.4764	0.8007	0.4649	0.049*	0.54
C6A	0.4225 (9)	0.7787 (6)	0.3768 (4)	0.0322 (18)	0.54
H6A1	0.3403	0.8210	0.3771	0.039*	0.54
H6A2	0.4702	0.7874	0.3397	0.039*	0.54
C7A	0.3742 (5)	0.6705 (4)	0.3827 (2)	0.0373 (14)	0.54
C14A	0.2879 (11)	0.6532 (10)	0.4336 (4)	0.0436 (17)	0.54
H14D	0.2193	0.7046	0.4372	0.065*	0.54
H14E	0.3464	0.6521	0.4678	0.065*	0.54
H14F	0.2404	0.5914	0.4297	0.065*	0.54
C15A	0.2798 (10)	0.6644 (10)	0.3284 (4)	0.0436 (17)	0.54
H15D	0.2465	0.5985	0.3237	0.065*	0.54
H15E	0.3329	0.6832	0.2945	0.065*	0.54
H15F	0.2007	0.7076	0.3330	0.065*	0.54
C8A	0.5004 (5)	0.5996 (4)	0.3788 (2)	0.0241 (11)	
H8A	0.5341	0.5913	0.4189	0.029*	
C5B	0.5252 (6)	0.8098 (4)	0.4280 (3)	0.0408 (14)	0.46
H5B1	0.5135	0.8403	0.3900	0.049*	0.46
H5B2	0.5237	0.8606	0.4576	0.049*	0.46
C6B	0.4056 (10)	0.7356 (7)	0.4391 (4)	0.0322 (18)	0.46
H6B1	0.3206	0.7706	0.4501	0.039*	0.46
H6B2	0.4316	0.6933	0.4713	0.039*	0.46
C7B	0.3742 (5)	0.6705 (4)	0.3827 (2)	0.0373 (14)	0.46
C14B	0.2512 (11)	0.6156 (11)	0.4076 (6)	0.0436 (17)	0.46
H14G	0.1848	0.6612	0.4238	0.065*	0.46
H14H	0.2836	0.5722	0.4377	0.065*	0.46
H14I	0.2065	0.5784	0.3773	0.065*	0.46
C15B	0.3436 (13)	0.7365 (10)	0.3366 (5)	0.0436 (17)	0.46
H15G	0.3090	0.7006	0.3035	0.065*	0.46
H15H	0.4278	0.7711	0.3259	0.065*	0.46
H15I	0.2731	0.7824	0.3492	0.065*	0.46
C9A	0.4633 (5)	0.4997 (4)	0.3580 (2)	0.0308 (13)	
C10A	0.4967 (6)	0.4694 (4)	0.2964 (2)	0.0347 (13)	
C11A	0.5131 (8)	0.5565 (5)	0.2563 (3)	0.0525 (17)	
H11C	0.5499	0.5342	0.2190	0.063*	
H11D	0.4209	0.5850	0.2492	0.063*	
C12A	0.6092 (6)	0.6342 (4)	0.2801 (2)	0.0316 (12)	
H12C	0.7019	0.6253	0.2629	0.038*	
H12D	0.5743	0.6974	0.2675	0.038*	
C13A	0.7716 (6)	0.7959 (4)	0.3355 (2)	0.0378 (14)	
H13D	0.8584	0.8179	0.3527	0.057*	
H13E	0.7070	0.8498	0.3328	0.057*	
H13F	0.7898	0.7704	0.2972	0.057*	
C16A	0.6231 (7)	0.4100 (5)	0.2982 (3)	0.0505 (17)	
H16D	0.6034	0.3497	0.3182	0.076*	
H16E	0.6962	0.4447	0.3186	0.076*	
H16F	0.6536	0.3961	0.2591	0.076*	
O9A	0.4112 (5)	0.4425 (3)	0.3907 (2)	0.0612 (14)	
O10A	0.3816 (5)	0.4186 (4)	0.2726 (2)	0.0631 (14)	
H10A	0.3630	0.3711	0.2933	0.095*	
Cl1A	0.91022 (13)	0.59664 (11)	0.32401 (6)	0.0384 (3)	
Cl2A	0.78988 (13)	0.54934 (10)	0.43211 (6)	0.0347 (3)	

### Atomic displacement parameters (Å^2^)

**Table:**

	*U*^11^	*U*^22^	*U*^33^	*U*^12^	*U*^13^	*U*^23^
C1	0.024 (2)	0.022 (2)	0.016 (2)	0.003 (2)	−0.0005 (18)	−0.005 (2)
C2	0.028 (2)	0.019 (2)	0.021 (2)	−0.001 (2)	0.003 (2)	0.001 (2)
C3	0.025 (2)	0.019 (2)	0.028 (2)	0.000 (2)	0.002 (2)	0.003 (2)
C4	0.024 (2)	0.025 (3)	0.033 (3)	−0.003 (2)	−0.007 (2)	−0.005 (2)
C5	0.026 (2)	0.029 (3)	0.037 (3)	0.005 (2)	−0.004 (2)	0.005 (3)
C6	0.034 (3)	0.026 (3)	0.025 (3)	0.005 (2)	0.002 (2)	0.000 (2)
C7	0.030 (2)	0.013 (2)	0.025 (3)	0.000 (2)	0.004 (2)	0.001 (2)
C8	0.025 (2)	0.016 (2)	0.017 (2)	0.003 (2)	0.0004 (19)	−0.004 (2)
C9	0.027 (3)	0.021 (3)	0.026 (3)	0.002 (2)	0.003 (2)	−0.010 (2)
C10	0.024 (2)	0.037 (3)	0.034 (3)	−0.003 (2)	−0.009 (2)	−0.008 (3)
C11	0.041 (3)	0.029 (3)	0.023 (3)	0.004 (3)	−0.012 (2)	−0.005 (2)
C12	0.033 (3)	0.029 (3)	0.015 (2)	0.007 (3)	0.000 (2)	−0.002 (2)
C13	0.030 (3)	0.032 (3)	0.044 (3)	0.001 (2)	0.010 (2)	0.004 (3)
C14	0.042 (3)	0.029 (3)	0.028 (3)	0.008 (3)	0.005 (2)	0.005 (3)
C15	0.033 (3)	0.025 (3)	0.035 (3)	0.006 (2)	0.003 (2)	−0.004 (2)
C16	0.032 (3)	0.035 (3)	0.051 (4)	0.008 (3)	−0.013 (3)	−0.003 (3)
O9	0.0342 (19)	0.030 (2)	0.045 (2)	−0.0094 (18)	0.0140 (18)	−0.0039 (19)
O10	0.038 (2)	0.044 (2)	0.062 (3)	−0.011 (2)	−0.016 (2)	−0.008 (2)
Cl1	0.0373 (7)	0.0254 (6)	0.0241 (6)	0.0032 (6)	0.0005 (5)	−0.0078 (6)
Cl2	0.0416 (7)	0.0243 (6)	0.0334 (7)	0.0054 (6)	−0.0017 (6)	0.0071 (6)
C1A	0.024 (2)	0.024 (3)	0.023 (3)	−0.003 (2)	0.002 (2)	−0.002 (2)
C2A	0.022 (2)	0.027 (3)	0.026 (3)	0.001 (2)	−0.001 (2)	0.001 (2)
C3A	0.025 (2)	0.019 (2)	0.026 (3)	−0.001 (2)	−0.004 (2)	0.003 (2)
C4A	0.048 (3)	0.022 (3)	0.029 (3)	−0.003 (2)	−0.003 (3)	0.002 (2)
C5A	0.044 (3)	0.027 (3)	0.052 (4)	0.003 (3)	0.005 (3)	−0.012 (3)
C6A	0.031 (4)	0.033 (4)	0.032 (4)	0.010 (4)	0.000 (3)	0.005 (4)
C7A	0.025 (3)	0.036 (3)	0.050 (4)	0.005 (3)	0.002 (3)	−0.002 (3)
C14A	0.030 (3)	0.060 (5)	0.040 (4)	0.015 (3)	0.008 (3)	0.009 (4)
C15A	0.030 (3)	0.060 (5)	0.040 (4)	0.015 (3)	0.008 (3)	0.009 (4)
C8A	0.022 (2)	0.022 (3)	0.028 (3)	0.000 (2)	0.000 (2)	−0.001 (2)
C5B	0.044 (3)	0.027 (3)	0.052 (4)	0.003 (3)	0.005 (3)	−0.012 (3)
C6B	0.031 (4)	0.033 (4)	0.032 (4)	0.010 (4)	0.000 (3)	0.005 (4)
C7B	0.025 (3)	0.036 (3)	0.050 (4)	0.005 (3)	0.002 (3)	−0.002 (3)
C14B	0.030 (3)	0.060 (5)	0.040 (4)	0.015 (3)	0.008 (3)	0.009 (4)
C15B	0.030 (3)	0.060 (5)	0.040 (4)	0.015 (3)	0.008 (3)	0.009 (4)
C9A	0.025 (3)	0.028 (3)	0.040 (3)	−0.004 (2)	0.005 (2)	−0.005 (3)
C10A	0.040 (3)	0.033 (3)	0.031 (3)	−0.008 (3)	−0.009 (2)	−0.010 (3)
C11A	0.063 (4)	0.048 (4)	0.046 (4)	−0.009 (4)	−0.021 (3)	0.004 (3)
C12A	0.035 (3)	0.036 (3)	0.024 (3)	−0.008 (3)	0.001 (2)	−0.004 (2)
C13A	0.040 (3)	0.032 (3)	0.041 (3)	−0.012 (3)	−0.002 (3)	0.011 (3)
C16A	0.065 (4)	0.047 (4)	0.040 (3)	0.011 (4)	0.000 (3)	−0.013 (3)
O9A	0.077 (3)	0.043 (3)	0.064 (3)	−0.033 (3)	0.043 (3)	−0.012 (2)
O10A	0.065 (3)	0.065 (3)	0.059 (3)	−0.025 (3)	−0.032 (3)	0.002 (3)
Cl1A	0.0246 (6)	0.0456 (8)	0.0450 (8)	0.0026 (6)	0.0110 (6)	0.0044 (7)
Cl2A	0.0345 (6)	0.0351 (7)	0.0345 (7)	0.0095 (6)	0.0003 (6)	0.0135 (6)

### Geometric parameters (Å, º)

**Table:**

C1—C8	1.521 (7)	C3A—C13A	1.523 (7)
C1—C2	1.521 (7)	C4A—C5B	1.513 (8)
C1—C12	1.525 (6)	C4A—C5A	1.513 (8)
C1—C3	1.547 (7)	C4A—H4A1	0.9900
C2—C3	1.493 (7)	C4A—H4A2	0.9900
C2—Cl1	1.764 (5)	C5A—C6A	1.614 (8)
C2—Cl2	1.770 (5)	C5A—H5A1	0.9900
C3—C4	1.515 (7)	C5A—H5A2	0.9900
C3—C13	1.529 (7)	C6A—C7A	1.586 (8)
C4—C5	1.539 (7)	C6A—H6A1	0.9900
C4—H4A	0.9900	C6A—H6A2	0.9900
C4—H4B	0.9900	C7A—C14A	1.473 (9)
C5—C6	1.522 (7)	C7A—C15A	1.566 (9)
C5—H5A	0.9900	C7A—C8A	1.574 (7)
C5—H5B	0.9900	C14A—H14D	0.9800
C6—C7	1.540 (7)	C14A—H14E	0.9800
C6—H6A	0.9900	C14A—H14F	0.9800
C6—H6B	0.9900	C15A—H15D	0.9800
C7—C15	1.523 (7)	C15A—H15E	0.9800
C7—C14	1.532 (7)	C15A—H15F	0.9800
C7—C8	1.599 (6)	C8A—C9A	1.517 (7)
C8—C9	1.515 (7)	C8A—C7B	1.574 (7)
C8—H8	1.0000	C8A—H8A	1.0000
C9—O9	1.222 (6)	C5B—C6B	1.574 (9)
C9—C10	1.528 (7)	C5B—H5B1	0.9900
C10—O10	1.422 (6)	C5B—H5B2	0.9900
C10—C11	1.528 (8)	C6B—C7B	1.628 (9)
C10—C16	1.533 (8)	C6B—H6B1	0.9900
C11—C12	1.536 (7)	C6B—H6B2	0.9900
C11—H11A	0.9900	C7B—C15B	1.448 (10)
C11—H11B	0.9900	C7B—C14B	1.529 (10)
C12—H12A	0.9900	C14B—H14G	0.9800
C12—H12B	0.9900	C14B—H14H	0.9800
C13—H13A	0.9800	C14B—H14I	0.9800
C13—H13B	0.9800	C15B—H15G	0.9800
C13—H13C	0.9800	C15B—H15H	0.9800
C14—H14A	0.9800	C15B—H15I	0.9800
C14—H14B	0.9800	C9A—O9A	1.214 (7)
C14—H14C	0.9800	C9A—C10A	1.535 (8)
C15—H15A	0.9800	C10A—O10A	1.432 (6)
C15—H15B	0.9800	C10A—C16A	1.478 (8)
C15—H15C	0.9800	C10A—C11A	1.543 (9)
C16—H16A	0.9800	C11A—C12A	1.532 (8)
C16—H16B	0.9800	C11A—H11C	0.9900
C16—H16C	0.9800	C11A—H11D	0.9900
O10—H10	0.8400	C12A—H12C	0.9900
C1A—C12A	1.515 (7)	C12A—H12D	0.9900
C1A—C2A	1.522 (7)	C13A—H13D	0.9800
C1A—C8A	1.535 (7)	C13A—H13E	0.9800
C1A—C3A	1.537 (7)	C13A—H13F	0.9800
C2A—C3A	1.491 (7)	C16A—H16D	0.9800
C2A—Cl1A	1.763 (5)	C16A—H16E	0.9800
C2A—Cl2A	1.769 (5)	C16A—H16F	0.9800
C3A—C4A	1.520 (7)	O10A—H10A	0.8400
			
C8—C1—C2	118.1 (4)	C13A—C3A—C1A	119.6 (4)
C8—C1—C12	114.4 (4)	C5B—C4A—C3A	114.1 (5)
C2—C1—C12	119.1 (4)	C5A—C4A—C3A	114.1 (5)
C8—C1—C3	116.1 (4)	C5A—C4A—H4A1	108.7
C2—C1—C3	58.2 (3)	C3A—C4A—H4A1	108.7
C12—C1—C3	119.8 (4)	C5A—C4A—H4A2	108.7
C3—C2—C1	61.8 (3)	C3A—C4A—H4A2	108.7
C3—C2—Cl1	121.0 (4)	H4A1—C4A—H4A2	107.6
C1—C2—Cl1	121.0 (3)	C4A—C5A—C6A	115.2 (5)
C3—C2—Cl2	118.7 (3)	C4A—C5A—H5A1	108.5
C1—C2—Cl2	120.5 (4)	C6A—C5A—H5A1	108.5
Cl1—C2—Cl2	108.0 (3)	C4A—C5A—H5A2	108.5
C2—C3—C4	118.9 (4)	C6A—C5A—H5A2	108.5
C2—C3—C13	119.6 (4)	H5A1—C5A—H5A2	107.5
C4—C3—C13	113.1 (4)	C7A—C6A—C5A	111.9 (6)
C2—C3—C1	60.0 (3)	C7A—C6A—H6A1	109.2
C4—C3—C1	117.0 (4)	C5A—C6A—H6A1	109.2
C13—C3—C1	118.5 (4)	C7A—C6A—H6A2	109.2
C3—C4—C5	112.3 (4)	C5A—C6A—H6A2	109.2
C3—C4—H4A	109.1	H6A1—C6A—H6A2	107.9
C5—C4—H4A	109.1	C14A—C7A—C15A	108.3 (7)
C3—C4—H4B	109.1	C14A—C7A—C8A	112.6 (6)
C5—C4—H4B	109.1	C15A—C7A—C8A	111.8 (5)
H4A—C4—H4B	107.9	C14A—C7A—C6A	113.1 (7)
C6—C5—C4	113.4 (4)	C15A—C7A—C6A	98.8 (6)
C6—C5—H5A	108.9	C8A—C7A—C6A	111.4 (5)
C4—C5—H5A	108.9	C7A—C14A—H14D	109.5
C6—C5—H5B	108.9	C7A—C14A—H14E	109.5
C4—C5—H5B	108.9	H14D—C14A—H14E	109.5
H5A—C5—H5B	107.7	C7A—C14A—H14F	109.5
C5—C6—C7	119.4 (4)	H14D—C14A—H14F	109.5
C5—C6—H6A	107.5	H14E—C14A—H14F	109.5
C7—C6—H6A	107.5	C7A—C15A—H15D	109.5
C5—C6—H6B	107.5	C7A—C15A—H15E	109.5
C7—C6—H6B	107.5	H15D—C15A—H15E	109.5
H6A—C6—H6B	107.0	C7A—C15A—H15F	109.5
C15—C7—C14	108.1 (4)	H15D—C15A—H15F	109.5
C15—C7—C6	106.5 (4)	H15E—C15A—H15F	109.5
C14—C7—C6	110.1 (4)	C9A—C8A—C1A	109.1 (4)
C15—C7—C8	113.4 (4)	C9A—C8A—C7A	114.3 (4)
C14—C7—C8	108.2 (4)	C1A—C8A—C7A	115.5 (4)
C6—C7—C8	110.5 (4)	C9A—C8A—C7B	114.3 (4)
C9—C8—C1	111.8 (4)	C1A—C8A—C7B	115.5 (4)
C9—C8—C7	110.3 (4)	C9A—C8A—H8A	105.7
C1—C8—C7	114.6 (4)	C1A—C8A—H8A	105.7
C9—C8—H8	106.6	C7A—C8A—H8A	105.7
C1—C8—H8	106.6	C4A—C5B—C6B	107.6 (6)
C7—C8—H8	106.6	C4A—C5B—H5B1	110.2
O9—C9—C8	120.8 (5)	C6B—C5B—H5B1	110.2
O9—C9—C10	119.4 (4)	C4A—C5B—H5B2	110.2
C8—C9—C10	119.8 (4)	C6B—C5B—H5B2	110.2
O10—C10—C9	109.9 (5)	H5B1—C5B—H5B2	108.5
O10—C10—C11	109.2 (4)	C5B—C6B—C7B	111.8 (6)
C9—C10—C11	110.3 (4)	C5B—C6B—H6B1	109.3
O10—C10—C16	106.4 (4)	C7B—C6B—H6B1	109.3
C9—C10—C16	108.9 (4)	C5B—C6B—H6B2	109.3
C11—C10—C16	112.1 (5)	C7B—C6B—H6B2	109.3
C10—C11—C12	115.2 (4)	H6B1—C6B—H6B2	107.9
C10—C11—H11A	108.5	C15B—C7B—C14B	116.2 (8)
C12—C11—H11A	108.5	C15B—C7B—C8A	121.0 (6)
C10—C11—H11B	108.5	C14B—C7B—C8A	108.2 (6)
C12—C11—H11B	108.5	C15B—C7B—C6B	106.6 (8)
H11A—C11—H11B	107.5	C14B—C7B—C6B	96.7 (7)
C1—C12—C11	115.1 (4)	C8A—C7B—C6B	104.7 (5)
C1—C12—H12A	108.5	C7B—C14B—H14G	109.5
C11—C12—H12A	108.5	C7B—C14B—H14H	109.5
C1—C12—H12B	108.5	H14G—C14B—H14H	109.5
C11—C12—H12B	108.5	C7B—C14B—H14I	109.5
H12A—C12—H12B	107.5	H14G—C14B—H14I	109.5
C3—C13—H13A	109.5	H14H—C14B—H14I	109.5
C3—C13—H13B	109.5	C7B—C15B—H15G	109.5
H13A—C13—H13B	109.5	C7B—C15B—H15H	109.5
C3—C13—H13C	109.5	H15G—C15B—H15H	109.5
H13A—C13—H13C	109.5	C7B—C15B—H15I	109.5
H13B—C13—H13C	109.5	H15G—C15B—H15I	109.5
C7—C14—H14A	109.5	H15H—C15B—H15I	109.5
C7—C14—H14B	109.5	O9A—C9A—C8A	120.1 (5)
H14A—C14—H14B	109.5	O9A—C9A—C10A	119.7 (5)
C7—C14—H14C	109.5	C8A—C9A—C10A	120.2 (5)
H14A—C14—H14C	109.5	O10A—C10A—C16A	112.2 (5)
H14B—C14—H14C	109.5	O10A—C10A—C9A	109.7 (5)
C7—C15—H15A	109.5	C16A—C10A—C9A	107.6 (5)
C7—C15—H15B	109.5	O10A—C10A—C11A	103.4 (5)
H15A—C15—H15B	109.5	C16A—C10A—C11A	111.9 (6)
C7—C15—H15C	109.5	C9A—C10A—C11A	112.0 (5)
H15A—C15—H15C	109.5	C12A—C11A—C10A	113.4 (5)
H15B—C15—H15C	109.5	C12A—C11A—H11C	108.9
C10—C16—H16A	109.5	C10A—C11A—H11C	108.9
C10—C16—H16B	109.5	C12A—C11A—H11D	108.9
H16A—C16—H16B	109.5	C10A—C11A—H11D	108.9
C10—C16—H16C	109.5	H11C—C11A—H11D	107.7
H16A—C16—H16C	109.5	C1A—C12A—C11A	116.0 (5)
H16B—C16—H16C	109.5	C1A—C12A—H12C	108.3
C10—O10—H10	109.5	C11A—C12A—H12C	108.3
C12A—C1A—C2A	118.0 (4)	C1A—C12A—H12D	108.3
C12A—C1A—C8A	115.4 (4)	C11A—C12A—H12D	108.3
C2A—C1A—C8A	117.7 (4)	H12C—C12A—H12D	107.4
C12A—C1A—C3A	119.6 (4)	C3A—C13A—H13D	109.5
C2A—C1A—C3A	58.4 (3)	C3A—C13A—H13E	109.5
C8A—C1A—C3A	115.9 (4)	H13D—C13A—H13E	109.5
C3A—C2A—C1A	61.4 (3)	C3A—C13A—H13F	109.5
C3A—C2A—Cl1A	119.7 (4)	H13D—C13A—H13F	109.5
C1A—C2A—Cl1A	120.5 (4)	H13E—C13A—H13F	109.5
C3A—C2A—Cl2A	120.6 (4)	C10A—C16A—H16D	109.5
C1A—C2A—Cl2A	120.6 (4)	C10A—C16A—H16E	109.5
Cl1A—C2A—Cl2A	108.0 (3)	H16D—C16A—H16E	109.5
C2A—C3A—C4A	119.1 (4)	C10A—C16A—H16F	109.5
C2A—C3A—C13A	118.4 (4)	H16D—C16A—H16F	109.5
C4A—C3A—C13A	112.2 (4)	H16E—C16A—H16F	109.5
C2A—C3A—C1A	60.3 (3)	C10A—O10A—H10A	109.5
C4A—C3A—C1A	118.3 (4)		

### Hydrogen-bond geometry (Å, º)

**Table:**

*D*—H···*A*	*D*—H	H···*A*	*D*···*A*	*D*—H···*A*
O10—H10···O9*A*	0.84	2.43	3.203 (7)	153
O10*A*—H10*A*···O9	0.84	2.11	2.945 (6)	173
C12—H12*B*···O10^i^	0.99	2.48	3.361 (7)	148

Symmetry code: (i) *x*−1/2, −*y*+1/2, −*z*+1.

## References

[bb1] Agilent (2014). *CrysAlis PRO*. Agilent Technologies Ltd., Yarnton, England.

[bb2] Altomare, A., Burla, M. C., Camalli, M., Cascarano, G. L., Giacovazzo, C., Guagliardi, A., Moliterni, A. G. G., Polidori, G. & Spagna, R. (1999). *J. Appl. Cryst.* **32**, 115–119.

[bb3] Blair, M., Forsyth, C. M. & Tuck, K. L. (2010). *Tetrahedron Lett.* **51**, 4808–4811.

[bb4] Farrugia, L. J. (2012). *J. Appl. Cryst.* **45**, 849–854.

[bb5] Groom, C. R., Bruno, I. J., Lightfoot, M. P. & Ward, S. C. (2016). *Acta Cryst.* B**72**, 171–179. 10.1107/S2052520616003954 PMC4822653 27048719

[bb6] Macrae, C. F., Bruno, I. J., Chisholm, J. A., Edgington, P. R., McCabe, P., Pidcock, E., Rodriguez-Monge, L., Taylor, R., van de Streek, J. & Wood, P. A. (2008). *J. Appl. Cryst.* **41**, 466–470.

[bb7] Murahashi, S.-I., Naota, T. & Hanaoka, H. (1993). *Chem. Lett.* pp. 1767–1770.

[bb8] Parsons, S., Flack, H. D. & Wagner, T. (2013). *Acta Cryst.* B**69**, 249–259. 10.1107/S2052519213010014 PMC3661305 23719469

[bb9] Salvador, J. A. R., Moreira, V. M., Hanson, J. R. & Carvalho, R. A. (2006). *Steroids*, **71**, 266–272. 10.1016/j.steroids.2005.11.002 16368121

[bb10] Sbai, F., Dakir, M., Auhmani, A., El Jamili, H., Akssira, M., Benharref, A., Kenz, A. & Pierrot, M. (2002). *Acta Cryst.* C**58**, o518–o520. 10.1107/s0108270102012283 12154317

[bb11] Sheldrick, G. M. (2015). *Acta Cryst.* C**71**, 3–8.

[bb12] Spek, A. L. (2009). *Acta Cryst.* D**65**, 148–155. 10.1107/S090744490804362X PMC2631630 19171970

[bb13] Tamura, Y., Yakura, T., Haruta, J.-I. & Kita, Y. (1985). *Tetrahedron Lett.* **26**, 3837–3840.

[bb14] Trost, B. M. & Fray, M. J. (1988). *Tetrahedron Lett.* **29**, 2163–2166.

[bb15] VanRheenen, V. & Shephard, K. P. (1979). *J. Org. Chem.* **44**, 1582–1584.

